# Effects of supplementary feeding on interspecific dominance hierarchies in garden birds

**DOI:** 10.1371/journal.pone.0202152

**Published:** 2018-09-05

**Authors:** Megan L. Francis, Kate E. Plummer, Bethany A. Lythgoe, Catriona Macallan, Thomas E. Currie, Jonathan D. Blount

**Affiliations:** 1 Centre for Ecology and Conservation, College of Life & Environmental Sciences, University of Exeter, Penryn Campus, Penryn, Cornwall, United Kingdom; 2 British Trust for Ornithology, The Nunnery, Thetford, Norfolk, United Kingdom; University of Tulsa, UNITED STATES

## Abstract

Individuals often differ in competitive ability, which can lead to the formation of a dominance hierarchy that governs differential access to resources. Previous studies of dominance have predominently focussed on within-species interactions, while the drivers of between-species competitive hierarchies are poorly understood. The increasing prevalence of predictable anthropogenic food subsidies, such as that provided by garden bird feeders, is likely to intensify between-species competition. However, the consequences for resource acquisition await detailed study, and in particular, whether competitive interactions are influenced by food quality is not known. Here, we examine competitive interactions amongst ten passerine species of birds utilising supplementary food sources of differing quality. We show that dominance rank is strongly predicted by body mass across species. Socially dominant, heavier species monopolised access to a food that had a relatively short handling time (sunflower hearts), spent longer on supplementary feeders, and pecked at lower rates. In contrast subordinate, lighter species were constrained to feed on a food that had a relatively long handling time (sunflower seeds with the hull intact). Our findings suggest that differences in body mass may result in between-species dominance hierarchies that place the heaviest species in the greatest control of supplementary feeding sites, gaining superior access to higher value foods. This may have important implications for the use of supplementary feeding as a conservation tool.

## Introduction

Competition between organisms exerts important selection pressures in ecological communities [1,2]. Across taxa, individuals commonly gain control of resources through aggressive contests or agonistic interactions including threat displays [3], vocalisations [4,5], and physical combat [6,7]. Such aggression can be energetically costly, while fighting may result in injury and even death [6,8,9,10,11]. However, the costs associated with aggressive behaviours can be avoided. In many taxa, repeated competitive interactions where one individual consistently wins over another, can lead to the formation of dominance hierarchies—ranking systems in which each animal is either dominant or subordinate to another [12]. Such hierarchies can decrease the number of aggressive interactions by allowing individuals to assess their chances of winning before engaging in a fight [13]. In addition, dominance hierarchies have been shown to influence access to resources; high-ranking individuals often exert greater control over food sources [14], secure a greater proportion of matings [15,16] and acquire better territories than their subordinate counterparts [17]. Therefore, position within a dominance hierarchy may have fitness consequences.

Although found both within- and between-species, the vast majority of research examining dominance hierarchies has been conducted in social groups of the same species (e.g. elephants and primates), where all individuals exploit and use resources in a similar way [18,19]. The structure of such within-species hierarchies can be determined by many factors including sex, age, previous experience, aggressive nature, and body mass—the latter having the greatest influence [20,21]. In contrast the determinants of rank in between-species dominance hierarchies remain poorly understood. Human activities have extensively modified biotic environments, in ways that are likely to influence the frequency of competitive interactions amongst species [22,23]. One such ecological perterbation is caused by the widespread practice of supplementary feeding of birds in domestic gardens and backyards [24]. Such supplementary feeding has increased rapidly since the 1970s; estimates suggest that up to 43% of households in the USA and 75% of households in the UK regularly feed wild birds [25,26]. Food availability is one of the main factors limiting bird populations, and thus supplementary feeding can enhance survival and reproductive success [27]. Yet the ecological effects of these huge subsidies remain poorly understood [28]. Food supplementation has created a valuable and defensible source of food in many ecological communities, which may lead to species having overlapping feeding ecology [28]. Due to the diverse range of body sizes across garden bird species, one might expect that body mass plays an important role in the structure of any between-species dominance hierarchy. Indeed, using citizen science data for the USA, Miller *et al*. [29] recently reported the existence of a between-species dominance hierarchy across species visiting supplementary food stations, which was largely explained by body mass. However, whether body mass differences amongst species may influence the ability to monopolise the most valuable foods has not been studied in this context before [30].

Here, we recorded competitive interactions amongst ten passerine species of garden birds in order to calculate a between-species dominance hierarchy. We then examined the importance of body mass as a determinant of rank in the hierarchy. Finally, we experimentally tested the effects of supplementary food value on between-species competition. Food value was manipulated by supplementing birds either with sunflower seed hearts, or with the same species of sunflower seed with the hull intact. The latter was deemed a relatively low value food, because it requires increased handling time by birds [31]. We hypothesized that: i) a between-species dominance hierarchy would be apparent at supplementary feeding sites; ii) dominance would increase with body mass across species; and iii) rank in the dominance hierarchy would determine differential access to supplementary food. Specifically, we predicted that relatively high-ranking species would command greater access to the higher value food, i.e. sunflower hearts, by competitively excluding subordinate species.

## Materials and methods

### Study site and experimental design

The study was conducted at the University of Exeter’s Penryn Campus [latitude/longitude: 50°10’N/5°07’W], in Cornwall, UK, from 7 November to 8 December 2016. Supplementary bird feeders with squirrel proof cages were positioned at three randomly selected sites along woodland edges and hedgerows. All of the sites bordered semi-improved or ploughed grassland and were located at least 150m apart. At each site, two cylindrical mesh bird feeders measuring 140mm diameter x 270mm tall were positioned hanging from branches approximately 1m from one another at a height of 2m above the ground. Ten days prior to data collection, each feeder was filled with a 50:50 (w/w) mixture of black sunflower seed and sunflower hearts (John E. Haith Ltd, Grimsby, UK) to allow habituation to the presence of supplementary food. At the beginning of the experiment one feeder at each site was randomly allocated to receive ‘low value’ food (black sunflower seeds) and the other ‘high value’ food (sunflower hearts). Supplementary food was provided *ad libitum*. The position of feeders at each site was systematically alternated between observations throughout the study to ensure that foraging behaviour was not influenced by the location of high and low value foods.

In total, the foraging behaviour of passerines was recorded for 110h over 29 days. Data collection took place over 4 hours each day (weather permitting) and was divided between two sessions: a morning session from sunrise to 2 h after sunrise, and an evening session from 2 h before sunset to sunset, to coincide with peak times of foraging activity. Supplementary feeding stations were observed by 2 researchers, with one individual observing and describing passerine activity, and the other recording observations onto datasheets. Observations were made using binoculars (Bushnell Natureview, 8 x 42; Bushnell, Surrey, UK), or by eye from a temporary hide (Riverside Outdoor Camo Protector 2 Pop Up Hide, 152 x 172 cm; Riverside Outdoor, Bolton, UK) placed approximately 5 m from a feeding site. During observations, the following parameters were measured for each passerine that visited the feeding station: species; food selection (low or high quality); the amount of time the bird spent on the feeder using a stopwatch (in seconds to 2 d.p.); the number of pecks made while on the feeder; and the outcome of any dominance interactions in which that individual was involved. For each species observed, an estimate of mean body mass was derived from Robinson *et al*. [32].

### Estimation of dominance rank

To determine whether there was a between-species dominance hierarchy operating at supplementary feeders, we observed dominance interactions between birds of different species. A dominance interaction was defined as any interaction between two individuals which resulted in one of the birds retreating from the food source [33]. In each interaction, an individual was classified as the ‘winner’ if it displaced another bird from the feeder through either aggressive behaviour, or non-physical intimidation. The other bird involved in the interaction was classified as the ‘loser’. Aggressive behaviour included threat displays, vocalisations and direct physical combat, while non-physical intimidation was recorded where an individual left a feeder within 1 s of another bird flying directy towards it. In total, 14 bird species were observed participating in dominance interactions across the three supplementary feeding sites. Species that were non-passerines, or those that were observed in fewer than 10 dominance interactions were excluded from the analysis, leaving ten passerine species for which a dominance hierarchy was estimated.

We used the randomised Elo-rating method recently described by Sánchez-Tójar *et al*. [34] to infer the dominance hierarchy and estimate associated uncertainty in species’ rankings, implemented using the “aniDom” R package [35]. The original Elo-rating method considers interactions sequentially and updates subjects’ ratings after each dominance interaction in which they participate, based on the difference in the actual outcome from the probablity of the higher-rated subject (e.g. individual, or species) winning [36,37]. Therefore it does not require a complete interaction matrix, where all subjects are observed competing with every other subject, and ratings can subsequently be used to infer dominance rank. The randomised Elo-rating procedure [34] is advantagous for the current study as we are interested in between-species interactions, rather than those occuring between individuals, and therefore the specific sequence of events in not important. Further, the randomisation (n = 1000) improves the estimation of species ranks and can also be used to generate associated 95% confidence intervals, which convey the level of uncertainty in the ranking of each species. In order to assess the robustness of the between-species dominance hierarchy estimation, (1) we calculated the repeatability score for species ranks based on the randomised Elo-ratings and (2) we estimated the degree of linearity, or ‘orderliness’, within the hierarchy using triangle transitivity (T_tri_) [34].

Data for species identities, mass and dominance rankings are available in the supporting information (S1 Dataset). Data for species interspecific interactions, food choice and foraging behaviour are also available in the supporting information (S2 Dataset).

### Statistical analyses

Linear regression was used to test whether dominance rank was associated with variation in body mass. Generalised linear mixed models (GLMMs) with a binomial error distribution, and including site as a random factor to account for pseudoreplication, were used to examine differences in food choice. Separate models were fitted using either species identity or species dominance rank as a predictor against food type, modelled as a binary response. In the model examining dominance, time of day was also included as a covariate to account for differences in foraging behaviour between morning and evening, along with its interaction term. To examine whether dominance influenced foraging behaviour, we modelled the amount of time birds spent on feeders per visit (‘time on feeder’) and peck rate as a function of dominance rank, respectively. Time on feeders was log-transformed and modelled using a linear mixed model (LMM) with gaussian errors, having checked for normality and heteroscedasdicity of the residuals. Peck rate was modelled as number of pecks, offset by the natural logarithm of time on feeder, using a GLMM with Poisson errors and including an observation-level random effect to account for overdispersion [38]. Both foraging behaviour models included dominance rank, food type and time of day as covariates, as well as two-way interactions involving dominance rank and site fitted as a random factor. To determine the significance of each fixed term, we removed the least significant terms (alpha = 0.05) from the maximal model by stepwise deletion until a minimal model was reached. All statistical analyses were performed using R, version 3.4.0.

#### Phylogenetic comparative analysis

The linear regression of dominance rank on body mass assumes that the data points being analysed are statistically independent. However, the phylogenetic relationships between species could potentially violate this assumption. For example, the fact that 10 species analysed here come from 6 different families with 3 species each coming from the Paridae and Muscicapidae families could introduce clustering into the data and lead to incorrect parameter estimates. We therefore ran a phylogenetic generalized least squares (PGLS) model that estimates the strength of body mass as a predictor of rank while incorporating knowledge about the phylogenetic relationships between species (S1 Methods).

## Results

### Between-species dominance hierarchy

**Fig 1:**
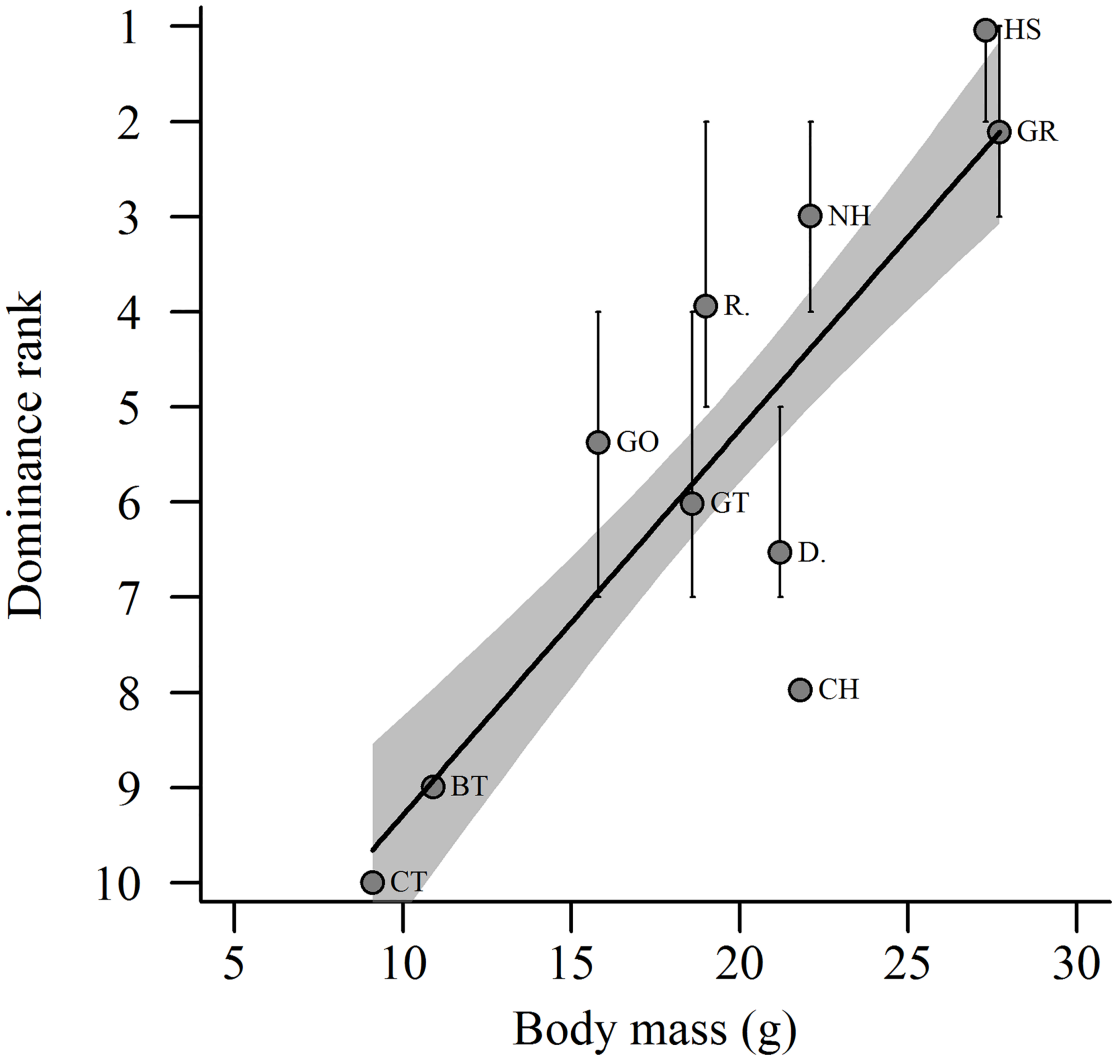
The relationship between body mass and dominance rank (±95% CI) of passerines at supplementary feeders. Dominance rank order has been reversed to illustrate increasing dominance along the y-axis, from least (rank = 10) to most (rank = 1) dominant. Species: (CT) coal tit, (BT) blue tit, (GO) goldfinch, (GT) great tit, (R.) robin, (D.) dunnock, (CH) chaffinch, (NH) nuthatch, (HS) house sparrow, (GR) greenfinch.

A total of 816 interspecific interactions were recorded at supplementary feeders, far exceeding the recommended sampling effort required to reliably estimate the dominance hierarchy between the ten species of interest [34]. House sparrow was estimated to be the most dominant species, while coal tit was the least dominant, with 95% confidence intervals indicating a high level of certainty in the species rank assignments, particularly amongst the lowest ranking species (Table 1). Further, species’ Elo-ratings were highly repeatable (r = 0.988) and the hierarchy was very transitive (Ttri = 1.00, p = 0.023), suggesting a high degree of confidence in the estimation of the hierarchy and that rank largely predicts the probability of winning an interaction. As predicted, dominance rank was found to be significantly positively associated with body mass across species (r2 = 0.698, F1,8 = 18.49, p = 0.003; Fig 1). However, examination of the confidences around species’ ranks and the fitted model suggests that chaffinches are considerably less dominant than expected given their body mass, while robins are more dominant (Fig 1). There was no obvious clustering of species by family (Fig 1) and the results of the PGLS analysis (β = -0.38, p = 0.007, r2 = 0.57) were extremely similar to the non-phylogenetic analyses, suggesting that the relationship between body mass and dominance rank was largely independent of species’ shared evolutionary histories.

**Table 1:** The species, body mass, and dominance rank of passerine birds observed participating in dominance interactions at supplementary feeding stations, in order of decreasing dominance. The 95% confidence range associated with each estimate of dominance rank is included as an a indicator of uncertainty, estimated using randomised Elo-ratings [34]. Species’ body masses were obtained from Robinson [32].

Species	Body mass (g)	Dominance rank	95% confidence range
House sparrow (*Passer domesticus*)	27.3	1.04	1–2
Greenfinch (*Carduelis chloris*)	27.7	2.12	1–3
Nuthatch (*Sitta europaea*)	22.1	3.00	2–4
Robin (*Erithacus rubecula*)	19.0	3.94	2–5
Goldfinch (*Carduelis carduelis*)	15.8	5.38	4–7
Great tit (*Parus major*)	18.6	6.02	4–7
Dunnock (*Prunella modularis*)	21.2	6.53	5–7
Chaffinch (*Fringilla coelebs*)	21.8	7.97	8–8
Blue tit (*Cyanistes caeruleus*)	10.9	9.00	9–9
Coal tit (*Periparus ater*)	9.1	10.00	10–10

### Food selection

**Fig 2:**
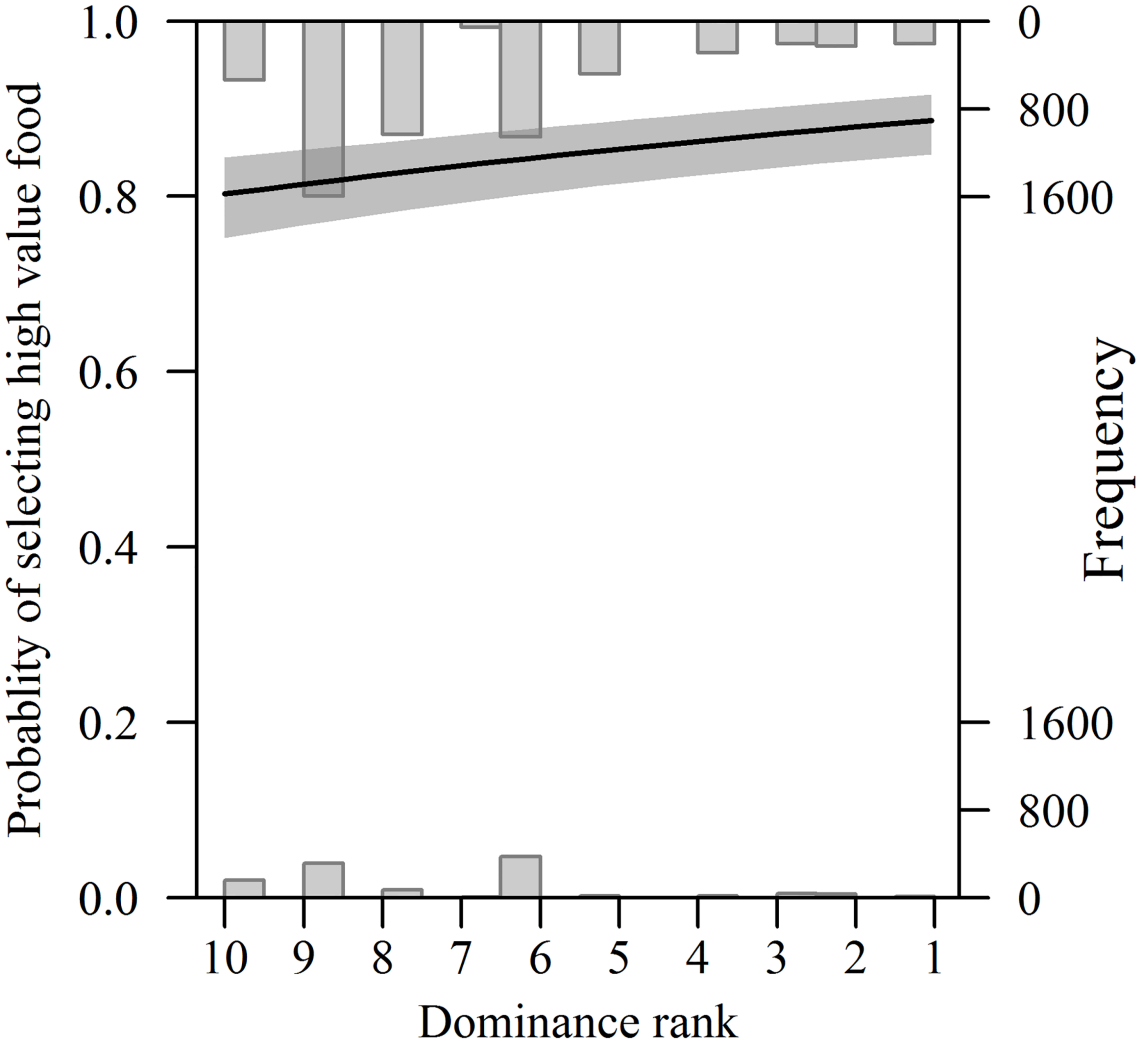
The relationship between dominance rank and food choice across ten passerine bird species. Dominance rank order has been reversed to illustrate increasing dominance along the x-axis, from least (rank = 10) to most (rank = 1) dominant. Histograms represent the distribution of the underlying data, and the line (± 95% CI, shaded) has been fitted from the minimum model.

Across the total survey period, 84.6% of birds (n = 5,696) were observed at feeders containing high value food (sunflower hearts), compared to only 15.4% (n = 1,033) at the low value (black sunflower seed) feeders. The probability of choosing the high value food varied significantly among species, with goldfinches most likely to choose sunflower hearts and great tits least likely (χ^2^_1_ = 407.73, *p* < 0.001). Moreover, food selection was influenced by dominance, with higher ranking species significantly more likely to be observed utilising sunflower hearts (χ^2^_1_ = 24.03, *p* < 0.001; Fig 2). Birds were also significantly more likely to choose the high value food before sunset than following sunrise (χ^2^_1_ = 7.16, *p* < 0.001), however, the effect of dominance on food choice was consistent across different times of day (dominance rank × time of day N.S.).

### Foraging tactics

**Fig 3:**
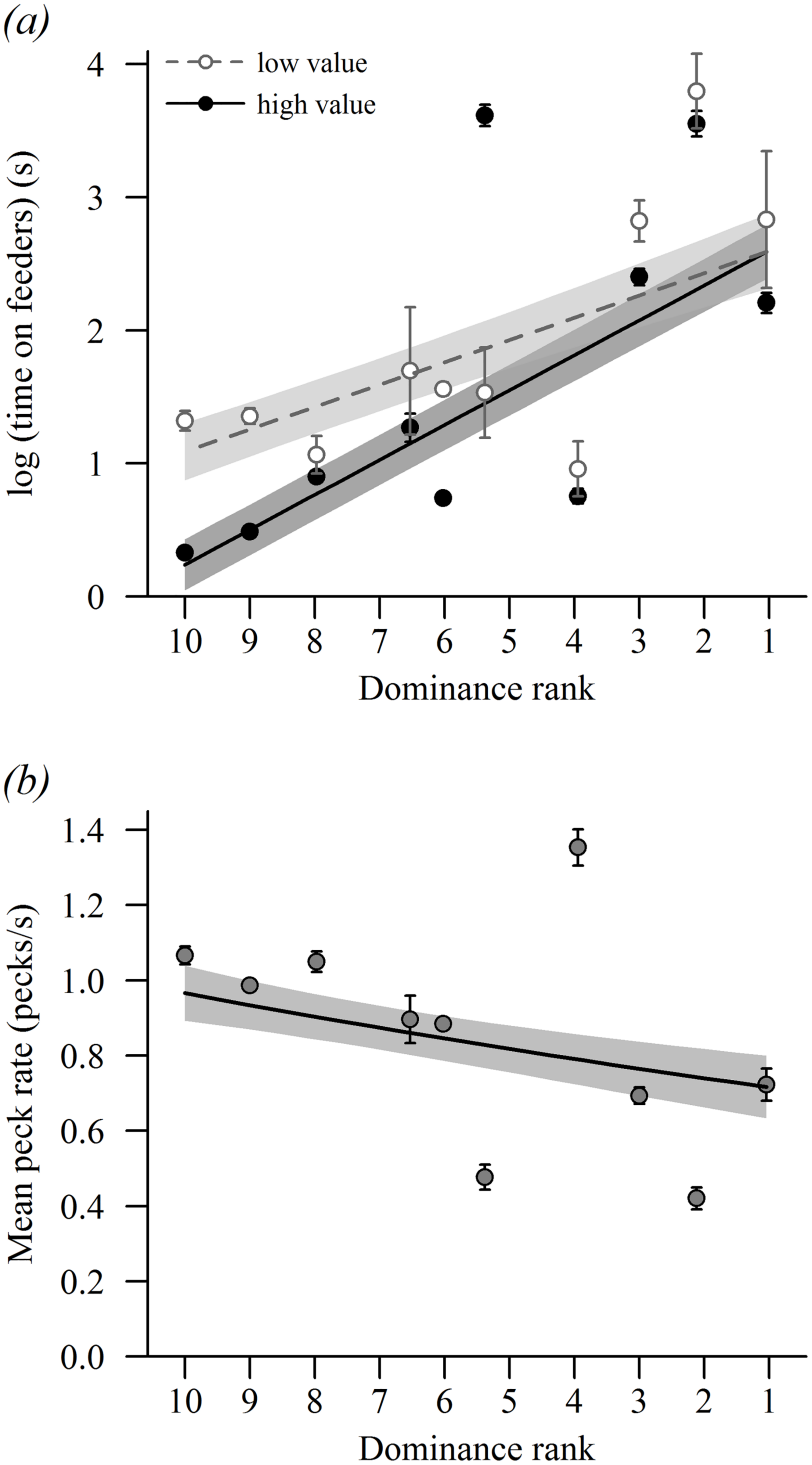
The effect of species dominance on foraging behaviour. Dominance rank order has been reversed to illustrate increasing dominance along the x-axis, from least (rank = 10) to most (rank = 1) dominant. (a) The relationship between the mean amount of time species spent on supplementary feeders (±SE) and dominance, as a function of food value. (b) The relationship between the mean peck rate of species (±SE) and dominance. Lines and 95% confidence intervals (shaded) are predicted from the minimum models.

Across species, variation in foraging behaviour was found to be significantly influenced by dominance (Fig 2). There was a significant positive relationship between species dominance rank and the mean amount of time spent on feeders (χ^2^_1_ = 238.25, *p* < 0.001), and this relationship further varied with food choice (food value × dominance: χ^2^_1_ = 30.34, *p* < 0.001; Fig 3a). While highly dominant species spent a similar length of time on both high and low value feeders, low ranking species were constrained to spend a significantly longer amount of time foraging at low value feeders (Fig 3a). In contrast, there was a significant negative relationship between peck rate and dominance rank (χ^2^_1_ = 11.61, *p* < 0.001; Fig 2b), which was consistent across food types (food value × dominance, N.S.). Peck rates were, however, higher on high value feeders (χ^2^_1_ = 27.62, *p* < 0.001) and before sunset (χ^2^_1_ = 9.61, *p* = 0.002; time of day × dominance, N.S.).

## Discussion

Based on direct observations of competitive interactions we provide evidence of a clearly defined ‘pecking order’ among ten small passerine bird species that commonly use supplementary feeders in gardens in the UK. A highly significant positive association between dominance rank and body mass across species suggests that the hierarchy is largely driven by variation in body mass or size. Our findings also suggest that a species’ position within the hierarchy can influence food choice and foraging tactics. More dominant species were significantly more likely to use the high value food option, sunflower hearts, compared with less dominant species. High-ranking species also tended to stay on supplementary feeders for longer periods of time and displayed lower peck rates, whereas low-ranking species remained on supplementary feeders for shorter periods of time and displayed higher peck rates.

We found a significant positive association between body mass and dominance across ten passerine species of birds that were observed to compete regularly at supplementary feeding sites. If closely related species have similarities in their foraging preferences and/or behaviour, it is possible that the dominance hierachy between species could result from shared evolutionary history. However, we found no evidence that the relationship was simply a consequence of phylogeny. Specifically, phylogenetic comparative analysis did not change the conclusion that species with greater body mass had increased competitive success and were more likely to displace smaller species from supplementary feeders. This clearly defined ‘pecking order’ is more surprising for the fact that the largest and the smallest species that we included in the dominance hierarchy differ in weight by only 18g (range: 9.1 g– 27.3 g). It would be interesting to investigate whether the observed relationship between body mass and dominance holds within-species, when accounting for individual variation in body mass. Within species, however, it can be difficult to separate the importance of body mass from additional, related factors that can influence dominance rank, for example, sex [39], age [40] or previous experience [41]. Our between-species study does not suffer from this limitation and suggests that, at least across these ten passerine species, rank-related differences in competitive ability were largely driven by variation in body mass or size. While we were not able to examine the potential influence of additional variables such as age and previous experience on dominance rank, such factors would not be expected to vary systematically across species.

It has previously been noted that socially dominant, heavier tits (Paridae) display a strong preference for higher quality natural food types, and exert greater control over natural foraging sites than subordinate, lighter tit species [14]. We therefore predicted that at focal supplementary feeding stations where intense between-species competition is expected, heavier and more dominant species would exhibit a preference for the highest value food that was available, while subordinate species would be constrained to utilise lower value food. Consistent with this expectation, we found that higher ranking, more dominant species were significantly more likely to be observed utilising sunflower hearts than lower ranking, less dominant species. Black sunflower seeds and sunflower hearts are of the same nutritional value to birds, but black sunflower seeds have the hull intact, and as such they require increased handling time [31]. Therefore, sunflower hearts are a relatively high value resource and it is not suprising that access to them was monopolised by more dominant species. Interestingly, for low ranking species the energetic benefit of foraging on sunflower hearts was apparently outweighed by the potential costs of aggressive interactions. There was considerably less between-species competiton at feeders containing black sunflower seeds, allowing subordinate species to gain greater access to food. In addition, birds were significantly more likely to choose the relatively high value food (sunflower hearts) before sunset than following sunrise. Because they require a relatively short handling time, sunflower hearts enable birds to gain a greater energetic benefit at a lower foraging cost. During winter, birds are likely to experience higher thermoregulatory costs overnight due to decreased ambient temperatures. Therefore, to enhance their likelihood of remaining in energy balance while minimising the time spent carrying large and costly fat reserves, they place particular emphasis on foraging for high value foods in the hours just before darkness [42,43]. Furthermore, consistent with the idea that there is a premium on maximising energy intake before roosting, peck rates were higher before sunset than after sunrise.

Peck rates were higher on the high value food (sunflower hearts). This presumably reflects the fact that sunflower seeds, the relatively low value food source, still have the hull of the seed present. Therefore, birds had to position each seed in their beak, crack open the coat, and remove the hull before ingesting [44]. There were significant relationships between dominance and peck rate, and between dominance and the amount of time birds spent on high value and low value foods. Dominant species tended to stay on feeders for longer periods of time and peck at lower rates than subordinate species. This finding is most likely due to the avoidance behaviour of subordinate birds. In competitive interactions, low-ranking individuals often lose a greater number of aggressive contests, incur more injuries, and suffer higher energetic costs than dominants [6,8,9,10,11]. As a result, subordinates may benefit from feeding quickly and reducing the amount of time spent on feeders to avoid costly interactions with dominant birds. However, while this may reduce the risk of aggression between individuals, it may impose other costs on low-ranking species. For example, although subordinates spend less time on supplementary feeders during each visit, it seems likely they must need to spend more time waiting in close proximity to supplementary feeding sites until feeding opportunities arise. Therefore, low-ranking species may have less time to invest in other important activities such as territory defence. A reduction in such activities may negatively impact the future survival and reproductive success of subordinate species [45,46]. In addition, the foraging tactics of subordinate birds may expose them to greater risk of predation. For example, in a within-species study of great tits, Krams [47] showed that the proportion of time that individuals spent scanning their surroundings before leaving the protection of the canopy to visit supplementary feeding stations was positively correlated with dominance rank. In relation to our findings across passerine species, it appears that individuals of low-ranking species moved quickly to avoid dominant birds, and consequently they may be expected to allocate less time to vigilance activities before visiting feeders. As such, smaller species may risk greater exposure to predation while larger, dominant species, avoid such dangers. Ultimately, the foraging tactics of low-ranking species may allow them to avoid engaging in costly interactions with dominant birds, however their alternative foraging strategies may impact fitness.

Our findings are some of the first to shed light on how bird species compete for access to supplementary food. In recent decades, bird feeding has become an increasingly popular activity in the UK [24] and throughout much of the world [48]. However, its ecological impacts are still poorly understood [28]. Temporal and spatial predictablity in the supply of anthropogenic food resources has been hypothesised to result in increasingly frequent competitive interactions amongst species [23], potentially leading to the formation of between-species dominance hierarchies [29]. Indeed, body mass differences amongst bird species can explain patterns of time spent at supplementary feeders in gardens and backyards in the USA [29]. Our findings add further support to the idea that between-species dominance hierarchies are an emergent property of supplementary feeding sites, and moreover we show that species differ in their ability to command access to high value foods. This could have important consequences for the conservation benefits of providing supplementary food. Indeed, Galbraith *et al*. [49,50] reported that introduced bird species outnumbered native species at supplementary food stations in New Zealand, suggesting that introduced species are more dominant. They speculated that native species may thus experience declines in abundance and local extinctions [49]. However, in contrast to the present study, they did not examine between-species competition or dominance hierarchies, nor investigate the association between body mass and dominance. Miller *et al*. [29] calculated a continent-wide dominance hierarchy incorporating species with non-overlapping distributions, using a large dataset of ‘citizen science’ records of birds at feeders in the USA. Our results, based on direct observations of competitive interactions at feeders, demonstrate that body mass differences amongst species strongly influence patterns of feeding behaviour and the ability to monopolise a higher quality resource. If the objective of supplementary feeding is to equally benefit all species of garden birds, then it would be useful to investigate the effects of a greater variety of food types, and feeder designs, on between-species dominance hierarchies.

In conclusion, our direct observations of between-species interactions at supplemental feeding sites showed that passerine species of birds with greater body mass were more dominant than smaller species. Heavier, more dominant species monopolised access to a relatively high value food (sunflower hearts), spent longer on feeders, and had a relatively low peck rate. In contrast lighter, subordinate species were constrained to spend a significantly longer amount of time foraging at low value feeders, and had a faster peck rate. Our results therefore suggest that differences in body mass may lead to the formation of between-species dominance hierarchies, leaving the heaviest species in the greatest control of higher value foods at supplemental feeding stations. This is likely to have important implications for the use of supplementary feeders as a conservation tool.

## Animal welfare note

This study was approved by the University of Exeter’s College of Life and Environmental Sciences (Penryn) Ethics Committee.

## Supporting information

S1 Methods Methods for phylogenetic comparative analysis, including the phylogeny used (Figure A). (DOCX)

S1 Dataset Data for species identities, mass and dominance rankings. (CSV)

S2 Dataset Data for species interspecific interactions, food choice and foraging behaviour. (CSV)
