# Supplementary material for: Effects of supplementary feeding on interspecific dominance hierarchies in garden birds
Source: PLoS One. 2018 Sep 5;13(9):e0202152. doi: 10.1371/journal.pone.0202152 (PMC6124729; doi:10.1371/journal.pone.0202152)
Supplement: S1 Methods — (DOCX) [file pone.0202152.s001.docx]

Supporting Material to accompany the article:

Effects of supplementary feeding on interspecific dominance hierarchies in garden birds

Megan L. Francis, Kate E. Plummer, Bethany A. Lythgoe, Catriona Macallan, Thomas E. Currie & Jonathan D. Blount

S1. Methods for phylogenetic comparative analysis

Phylogenetic generalized least squares (PGLS) analyses were conducted in the R package CAPER with the phylogenetic signal parameter (“lambda”) set at a value of 1, i.e. making the assumption for the purpose of this analysis that trait values are well-predicted by the phylogenetic relationships between species [1]. Phylogenetic trees were taken from the supertree produced by Davis & Page [2]. PGLS requires that the input phylogeny has branch lengths indicating the evolutionary distance between species. However, the published version of the supertree does not include information about branch lengths, therefore all branch lengths were set to take an arbitrary value of 1. The Eurasian nuthach (*Sitta europaea*) was not included in the supertree and so a replacement species with the same relative relationships to the other species in this analysis, the Himalayan nuthatch (*Sitta himalayensis*), was used instead. Species not included in this analysis were pruned from the supertree to leave the phylogeny shown in Figure A.


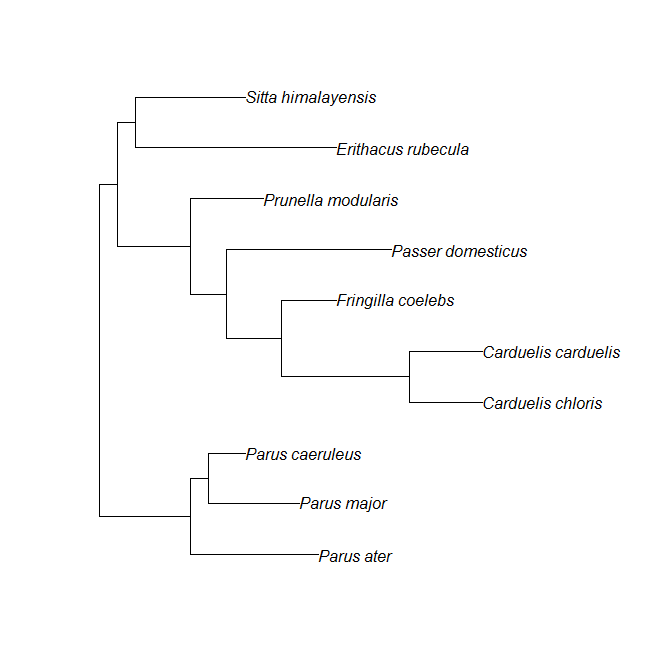


Figure A. Phylogenetic relationships between 10 species used in the analysis based on the supertree produced by Davis & Page [2].

References

1. Orme D, Freckleton R, Thomas G, Petzoldt T, Fritz S, et al. (2012) CAPER: Comparative Analyses of Phylogenetics and Evolution in R, version 0.5. <http://cran.r-project.org/web/packages/caper/index.html>.

2. Davis KE, Page RDM (2014) Reweaving the Tapestry: a Supertree of Birds. PLoS Currents 6: ecurrents.tol.c1af68dda67c999ed999f991e994b992d992df997a908e.
